# Ultrasonographic Diagnosis of Thoracic Outlet Syndrome Secondary to Brachial Plexus Piercing Variation

**DOI:** 10.3390/diagnostics7030040

**Published:** 2017-07-04

**Authors:** Vanessa Leonhard, Gregory Caldwell, Mei Goh, Sean Reeder, Heather F. Smith

**Affiliations:** 1 Department of Osteopathic Manipulative Medicine, Arizona College of Osteopathic Medicine, Midwestern University, Glendale, AZ 85308, USA; vleonhard26@midwestern.edu (V.L.); gcaldwell36@midwestern.edu (G.C.); SReede@midwestern.edu (S.R.); 2 Arizona College of Osteopathic Medicine, Midwestern University, Glendale, AZ 85308, USA; mgoh24@midwestern.edu; 3 Department of Anatomy, Midwestern University, Glendale, AZ 85308, USA; 4 School of Human Evolution and Social Change, Arizona State University, Tempe, AZ 85287, USA

**Keywords:** anatomical variation, brachial plexus, superior trunk, middle trunk, anterior scalene muscle, neurogenic thoracic outlet syndrome, ultrasound, provocative testing

## Abstract

Structural variations of the thoracic outlet create a unique risk for neurogenic thoracic outlet syndrome (nTOS) that is difficult to diagnose clinically. Common anatomical variations in brachial plexus (BP) branching were recently discovered in which portions of the proximal plexus pierce the anterior scalene. This results in possible impingement of BP nerves within the muscle belly and, therefore, predisposition for nTOS. We hypothesized that some cases of disputed nTOS result from these BP branching variants. We tested the association between BP piercing and nTOS symptoms, and evaluated the capability of ultrasonographic identification of patients with clinically relevant variations. Eighty-two cadaveric necks were first dissected to assess BP variation frequency. In 62.1%, C5, superior trunk, or superior + middle trunks pierced the anterior scalene. Subsequently, 22 student subjects underwent screening with detailed questionnaires, provocative tests, and BP ultrasonography. Twenty-one percent demonstrated atypical BP branching anatomy on ultrasound; of these, 50% reported symptoms consistent with nTOS, significantly higher than subjects with classic BP anatomy (14%). This group, categorized as a typical TOS, would be missed by provocative testing alone. The addition of ultrasonography to nTOS diagnosis, especially for patients with BP branching variation, would allow clinicians to visualize and identify atypical patient anatomy.

## 1. Introduction

Neurogenic thoracic outlet syndrome (nTOS) is a neurologic impingement syndrome that is notoriously difficult to diagnose in the clinical setting [1,2]. There are vascular and neurogenic forms of thoracic outlet syndrome (TOS), with nTOS being the most common and comprising over 90% of cases [3]. The arterial type, affecting the subclavian artery, is more concretely diagnosable by traditional provocative tests [1], as these directly evaluate the radial pulse. Adson’s [4], Wright’s, and Costoclavicular tests utilize the classic relationship of the subclavian artery and the branches of the brachial plexus to identify specific sites of neurovascular impingement (Table 1). These tests diagnose compression at three distinct sites: within the interscalene space, deep to the pectoralis minor tendon, and between the first rib and clavicle. Adson’s test evaluates the passage of the brachial plexus trunks and subclavian artery as they pass through the interscalene space between the anterior and middle scalene muscles and relies on change in radial pulse due to compression of the subclavian artery between those muscles [4].

However, recent studies have determined that in individuals with brachial plexus branching variants [5,6,7], the nerve branches may be impinged within the anterior scalene muscle belly, while the subclavian artery travels unencumbered. These structural variants undermine traditional provocative testing by violating the assumption of concomitant impingement of the neurologic and arterial structures. Cadaveric study has uncovered a significant percentage of variation of the brachial plexus trunks at this level [5,6,7]. In the most prevalent variation, the superior piercing variation, the superior trunk (or its components: the anterior rami of C5 and C6) pierces the anterior scalene muscle. A multiple piercing variant was observed as well, in which the superior and middle trunks both pass independently through the anterior scalene muscle [7]. Together, these piercing variants have been found in with up to 48% of individuals deviating from the classic anatomical arrangement [7]. In patients with a structural variation in which portions of the brachial plexus course through the anterior scalene, this test would be falsely negative. These structures create increased diagnostic difficulty as the current diagnostic standard in the primary care setting relies on identical passage of the artery and plexus through this space.

TOS most commonly presents with neurological symptoms of pain and paresthesias, recorded in 98–100% of TOS patients (e.g., [8,9,10]). Symptoms are primarily located in the proximal arm (88%), shoulder (88%), and all five digits (58%) [3]. These nonspecific findings are associated with numerous forms of pathology in the upper extremity and the cervical region [11,12,13,14]. Similarly, the current definitions of TOS vary among clinicians. One study determined that surgeons are 100 times more likely to diagnose TOS than neurologists [15]. In general, current diagnostic criteria typically require that the provocative tests cause vascular change at the radial artery, regardless of symptoms. Disputed, or non-specific TOS is quite common, occurring when patients present with TOS-like symptoms, but do not meet the currently accepted diagnostic standards and, therefore, lack a definitive explanation for their symptoms (e.g., [16]). Individuals with variations from classic anatomical relationships, such as the superior piercing variation, are likely to present in this manner and remain without clear diagnosis or treatment strategy. To achieve more comprehensive diagnosis and plan of care, ultrasonography may offer a means to visualize the anatomy of the thoracic outlet, identify clinically relevant variations, and provide a distinct diagnosis for these patients.

Previous studies into the efficacy of provocative testing indicated that up to 60% of asymptomatic patients experienced vascular compromise during testing, a diagnostic false positive for TOS [17,18,19]. Considering the high prevalence of variation within the brachial plexus trunks, and associated lack of vascular change, the Adson’s test also has a high propensity for false negatives, up to 10%. One explanation for these results may be that a subset of patients presenting with nTOS symptoms, may be variant in the relationships of the thoracic outlet structures. Ultrasound imaging may be able to visualize these brachial plexus variants, therefore providing a diagnosis for those who would otherwise be missed by provocative testing.

Recently, new sets of criteria for diagnosing TOS have been proposed [20,21,22]. The Consortium for Outcomes Research and Education on Thoracic Outlet Syndrome proposed a preliminary set of detailed diagnostic TOS criteria [20,22]. This comprehensive list is an invaluable resource. However, while the study acknowledges that scalene muscular variation may exist, the implication is that such variation is rare and “too small to be detected by standard imaging tests, such as plain X-rays, CT or MRI scanning” and can, therefore, only be assessed at the time of surgery [22]. A second set of updated TOS reporting standards were recently published by the Society for Vascular Surgery [21] which include: symptoms of pathology at the thoracic outlet, symptoms of nerve compression, the absence of other pathology potentially explaining the symptoms, and a positive scalene muscle injection test. While useful, these standards do not account for common structural variation at the thoracic outlet. The criteria presume that “the brachial plexus and subclavian artery traverse the same spaces” [21] (p. e25). Therefore, patients with brachial plexus branching variants would lack the first diagnostic criterion because they have no pathology present at the thoracic outlet, only a common anatomical variation. Another potential limitation of this set of standards is that it requires the use of scalene muscle injections, which may not be accessible to a primary care physician in the clinic. Recently, electrodiagnostic methods have been developed which can result in more objective neurological findings regarding TOS (e.g., [23]). However, for the average primary care physician, this technology may not be available in the clinic and, thus, the use of these techniques is primarily relegated to specialists.

Given the recent discovery that piercing variants in the brachial plexus are quite common [5,6,7], and may predispose these individuals to nTOS, this study seeks to empirically evaluate the proposed association between brachial plexus piercing variants and nTOS symptoms. We also aim to determine the applicability of ultrasonography (US) for increasing the efficacy of clinical diagnosis over traditional provocative testing alone, especially for cases of nTOS secondary to BP variation.

## 2. Materials and Methods

### 2.1. Cadaveric Data

The cadaveric investigation assessed proximal brachial plexus branching variation in 95 cadaveric brachial plexus specimens (44 male, 51 female) from the gross anatomy teaching laboratories at Midwestern University. Cadavers were obtained for teaching purposes from the National Body Donation Program (St. Louis, MO, USA). The neck and shoulder of each cadaver were dissected bilaterally following *Grant’s Dissector* 16th ed. [24] to thoroughly reveal the brachial plexus. The inferior and lateral borders of the anterior scalene muscle were defined, and the position of the roots, trunks, and cords of the brachial plexus in relation to the scalene muscles was determined and documented. For each cadaveric specimen, the type(s) of brachial plexus branching variation and sidedness of each variant was recorded. Each specimen was evaluated by two members of the research team to confirm the assessment, and photo-documented for future confirmation. *t*-tests were then performed in SPSS 19 (IBM Corp., Chicago, IL, USA) to assess whether significant differences existed in the frequency of brachial plexus variants between the sexes.

### 2.2. Ultrasonography

Twenty-two volunteer student subjects were recruited from Midwestern University in Glendale, AZ, USA. Screening began with a comprehensive questionnaire covering pertinent past medical history, trauma history, and symptoms of neurovascular pathology in the upper extremity. Subjects were then tested using standard nTOS provocative testing, including Adson’s, Costoclavicular, and Hyperabduction/Wright tests (Table 1). Additional tests to rule out other upper extremity neurogenic conditions were also utilized, including Carpal Compression and Modified Spurling’s tests (Table 1). Any changes in radial arterial pulse or reproduction of symptoms were noted. The protocol for this study was approved by Midwestern University’s Institutional Review Board (IRB AZ#885, 9 March 2016).

Following completion of provocative testing, participants underwent ultrasound (US) study of the lateral neck using a Sonoscape S8 portable ultrasound unit. Starting with the US probe in the supraclavicular fossa, imaging was completed up to the angle of the mandible in both neutral and Adson’s test position bilaterally. A visual scan was conducted to identify the three hypoechoic trunks with a hyperechoic fascial separation from the anterior and middle scalene muscles. A lack of visible hyperechoic fascia between the anterior scalene and any of the trunks indicated a brachial plexus piercing variant. The branching pattern of the proximal brachial plexus, and the relationship of the trunks to the scalene muscles were documented bilaterally. Still images and video capture were used to record the anatomy for future verification. Researchers conducting US were blind to the results of the questionnaire and provocative testing. Ultrasound results were confirmed with a board certified radiologist.

To determine whether statistically significant correlations exist between reported TOS symptoms, brachial plexus branching variants (as identified by ultrasound) and provocative test results, a series of statistical analyses were conducted in SPSS 19 (IBM Corp.). Brachial plexus branching (ultrasound) results were coded as: piercing versus classic anatomy. Provocative test results were coded as separate variables for: any positive pulse or symptom reproduction during test, pulse response, and symptoms reproduced. Due to the bilaterally asymmetrical nature of brachial plexus branching, the left and right sides for each subject were considered separately. Bivariate correlation analyses were then performed between TOS symptoms and: brachial plexus variation, and each of the provocative test results. Partial correlation analyses were subsequently conducted between TOS symptoms and provocative test results while controlling for brachial plexus variation.

## 3. Results

### 3.1. Cadaveric Results

**Figure 1:**
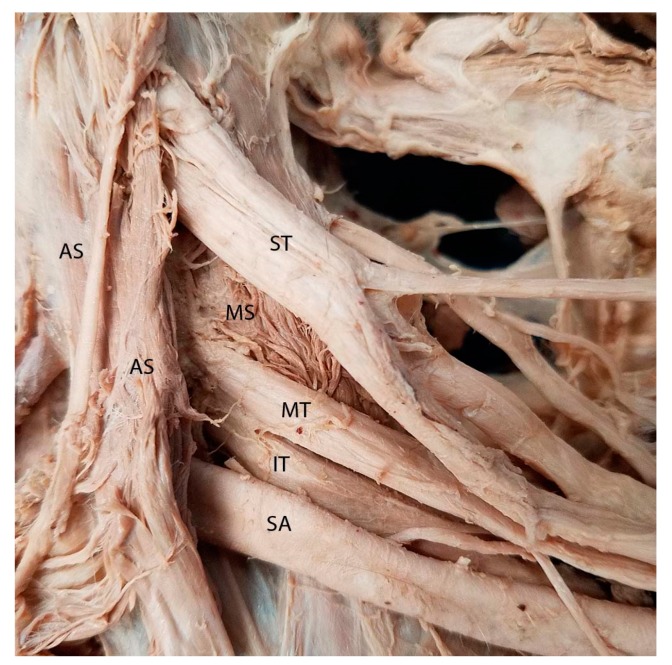
Cadaveric photo illustrating the classic anatomical relationship between the scalene musculature and the trunks of the brachial plexus. In this arrangement, the superior, middle, and inferior trunks of the brachial plexus all course between the anterior and middle scalene muscle, through the interscalene gap. AS = anterior scalene; IT = inferior trunk; MS = middle scalene; MT = middle trunk; SA = subclavian artery; ST = superior trunk.

**Figure 2:**
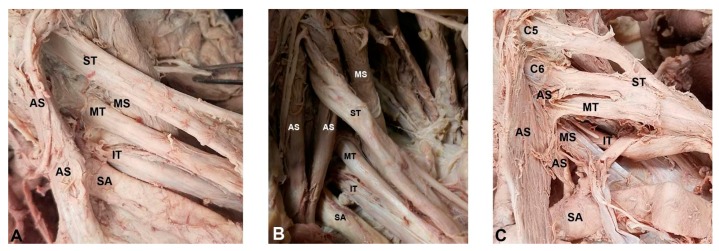
Anatomical relationships between the proximal brachial plexus and scalene musculature identified in the cadaveric component of the present study: (**A**) classic anatomical relationship between the brachial plexus and anterior scalene muscle. Superior, middle, and inferior trunks of the brachial plexus travel with the subclavian artery through the interscalene gap, between the anterior and middle scalene muscles; and (**B**) the superior piercing variant. The superior trunk of the brachial plexus pierces the anterior scalene muscle; and (**C**) the multiple piercing variant. The superior and middle trunks of the brachial plexus pierce the anterior scalene muscle. AS = anterior scalene; C5 = anterior ramus of C5; C6 = anterior ramus of C6; IT = inferior trunk; MS = middle scalene; MT = middle trunk; SA = subclavian artery; ST = superior trunk.

In the cadaveric sample (*n* = 95 plexi), brachial plexus branching variants were extremely common (Table 2 and Table S1). Only 32 brachial plexi (33.7%) were found to possess the “classic” anatomical pattern in which all three trunks of the brachial plexus course through the interscalene triangle (Figure 1). In the sample, 63 variations from the classic anatomical pattern were observed (Table 2, Figure 2B,C), such that 66.3% of the sample did not display the classic relationship between the scalene musculature and proximal brachial plexus. These variations can be classified into four categories: superior piercing (54.7%), multiple piercing (4.2%), C5 piercing (3.2%), and C5 anterior variant (3.4%). In each variant, one or more components of the brachial plexus course(s) in a position of relative vulnerability where it is more likely to become impinged. The most common clinically-relevant variants are depicted in Figure 2. The variant anatomy occurred more frequently in male cadavers than in females (74.5% vs. 56.8%); however, the *t*-test indicated that these differences between the sexes did not reach the statistical threshold for significance (*t* = −1.83, *p* = 0.07).

### 3.2. Ultrasonographic Results

**Figure 3:**
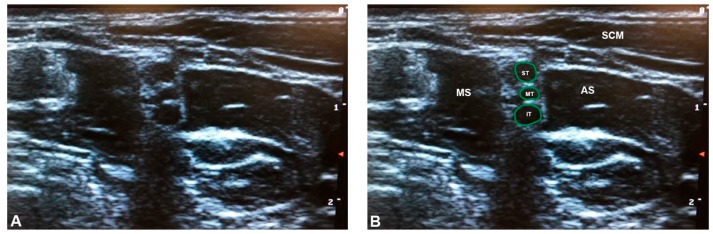
The classic brachial plexus anatomy identified on ultrasound: (**A**) unlabeled; and (**B**) labeled. Note that the superior, middle, and inferior trunks are clearly separated from the anterior and middle scalene muscles by hyperechoic fascial planes. AS = anterior scalene; IT = inferior trunk; MS = middle scalene; MT = middle trunk; SCM = sternocleidomastoid; ST = superior trunk. The green outlines demarcate the trunks of the brachial plexus.

**Figure 4:**
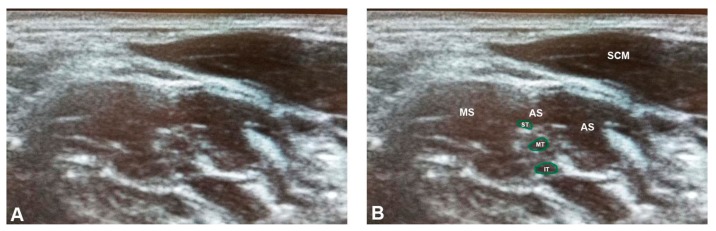
The superior piercing variant, the most common brachial plexus variant, identified using ultrasonography in the present study: (**A**) unlabeled; and (**B**) labeled. Note that the superior trunk is not separated from the anterior scalene in this condition, visible as a lack of hyperechoic fascia. AS = anterior scalene; IT = inferior trunk; MS = middle scalene; MT = middle trunk; SCM = sternocleidomastoid; ST = superior trunk. The green outlines demarcate the trunks of the brachial plexus.

**Figure 5:**
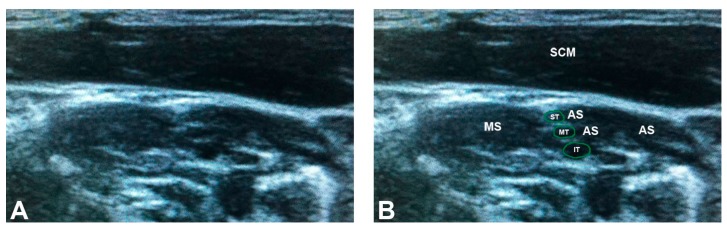
The multiple piercing variant, identified using ultrasonography in the present study: (**A**) unlabeled; and (**B**) labeled. Note that the superior and middle trunks are not separated from the anterior scalene in this condition, visible as a lack of hyperechoic fascia. AS = anterior scalene; C5 = anterior ramus of C5; C6 = anterior ramus of C6; IT = inferior trunk; MS = middle scalene; MT=middle trunk; SCM = sternocleidomastoid; ST= superior trunk. The green outlines demarcate the trunks and roots of the brachial plexus.

**Figure 6:**
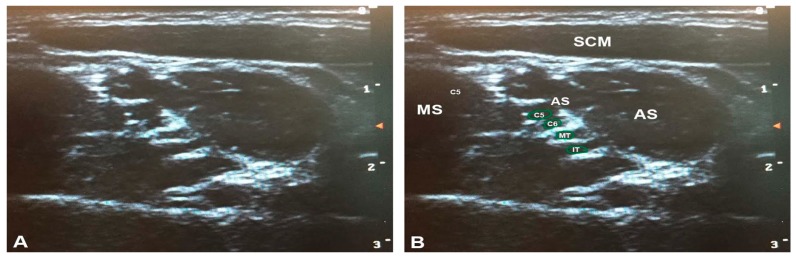
The C5 piercing variant, identified using ultrasonography in the present study: (**A**) unlabeled; and (**B**) labeled. Note that the C5 anterior ramus is not separated from the anterior scalene in this condition, visible as a lack of hyperechoic fascia. AS =anterior scalene; C5 = anterior ramus of C5; C6 = anterior ramus of C6; IT = inferior trunk; MS = middle scalene; MT = middle trunk; SCM = sternocleidomastoid; ST = superior trunk. The green outlines demarcate the trunks and roots of the brachial plexus.

**Figure 7:**
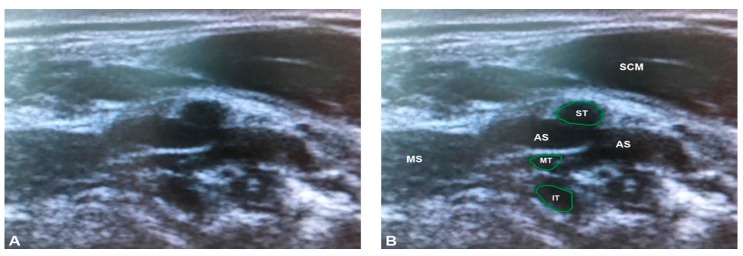
The anterior variant, identified using ultrasonography in the present study: (**A**) unlabeled; and (**B**) labeled. Note that the superior trunk courses superficial to the anterior scalene muscle in this condition. AS = anterior scalene; IT = inferior trunk; MS = middle scalene; MT = middle trunk; SCM = sternocleidomastoid; ST = superior trunk. The green outlines demarcate the trunks of the brachial plexus.

In the ultrasonographic screening sample, 79.5% of the sample was found to possess classic brachial plexus anatomy (Figure 3), while a total of nine brachial plexus branching variants were identified (20.5%) (Table 3 and Table S2). Eight of these were classified as piercing variants, in which portions of the brachial plexus coursed through the anterior scalene muscle (18.2%). The most common variation was the superior piercing variant (*n* = 4; 9.1%) (Figure 4), followed by the multiple piercing variant (*n* = 3; 6.8%) (Figure 5), and C5 piercing variant (*n* = 1; 2.3%) (Figure 6). There was also one example of a non-piercing anterior variant (2.3%) (Figure 7).

**Figure 8:**
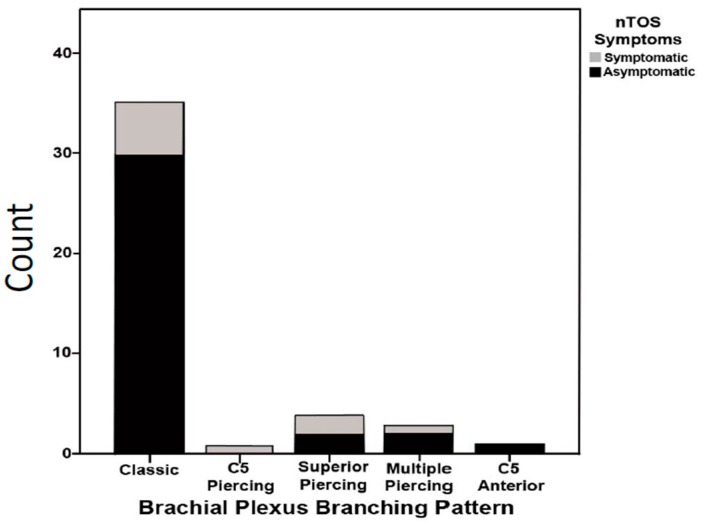
Frequency of brachial plexus branching patterns identified via ultrasonography in the screening portion of this study, and association with reported symptoms consistent with neurogenic thoracic outlet syndrome (nTOS). Symptomatic subjects are indicated in grey, while asymptomatic subjects are indicated in black. The percentages of symptomatic subjects are significantly higher in the piercing variant categories than in the normal sample of subjects with classic brachial plexus anatomy.

Of the eight instances of piercing variants, four were found in association with nTOS symptoms (50%), in contrast to five symptomatic instances in the 38 normal plexuses (13.9%) (Figure 8). The correlation analysis revealed a statistically significant correlation between brachial plexus piercing variants and nTOS symptoms (*r* = 0.345, *p* = 0.022). We classified these patients as presenting with atypical TOS, in which the nTOS symptoms are caused by impingement of the brachial plexus within the anterior scalene muscle belly, rather than in the interscalene gap. The other four atypical brachial plexus variant individuals may still be at increased risk for TOS based upon their anatomy; however, at the time of this study, they were asymptomatic. The sensitivity, specificity, positive predictive values and negative predictive value were determined to be 44%, 88.6%, 50%, and 86.1%. These values were determined using patient reported symptoms as a surrogate gold standard. The criteria for included symptoms was based upon common characteristics of nTOS described in the current literature. This surrogate was selected because it represents the patient population that would present for diagnosis and treatment in a clinical setting.

Across the entire sample, there were nine total instances of reported symptoms consistent with nTOS (20.5%), which is consistent with the presentations common to TOS as documented in previous studies [9,10]. Given that the student population is predicted to be at higher risk for neurogenic symptoms due to hypertonicity of the cervical musculature, a minor increase in cases was expected in this study. Within the full clinically symptomatic group, three subjects (33.3%) had positive Adson’s tests, while two had positive Wright tests (22.2%) (Table 4). These individuals represent the subset of Typical TOS in which the compression occurs between hypertonic anterior and middle scalene muscles.

**Figure 9:**
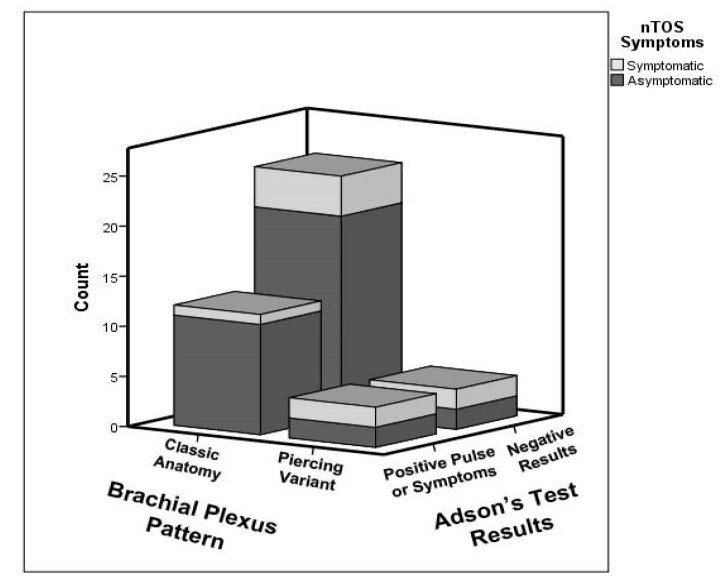
Summary of brachial plexus pattern and Adson’s Test results as associated with nTOS symptoms across the full screening sample. Symptomatic subjects are indicated in light grey, while asymptomatic subjects are indicated in dark grey. The percentage of individuals with nTOS symptoms was significantly higher among the brachial plexus piercing variant subjects (50%) than in the subjects with classic brachial plexus anatomy (13.9%), but rates of correct diagnostic identification with Adson’s Test were slightly lower (50% in piercing variants vs. 61.1% in classic).

Within the group of participants who denied symptoms on questionnaire (*n* = 35), the provocative tests demonstrated substantial potential for false positives. There were seventeen instances in which a positive result was found for at least one of the three provocative tests without a history of symptoms (48.6% false positives). Of these 17 overall positives, 13 were positive Adson’s tests (Figure 9). The correlation analyses revealed no statistically significant correlations between nTOS symptoms and any of the provocative tests (for all results, pulse, and symptoms). The partial correlation analysis controlling for brachial plexus variation also revealed no significant correlation between nTOS symptoms and the provocative tests.

## 4. Discussion

### 4.1. Anatomical Variation Observed

The findings from this study support the hypothesis that some cases of disputed TOS may result from brachial plexus variations in which the roots or trunks of the plexus course through the anterior scalene muscle belly, becoming impinged. This phenomenon is similar to piriformis syndrome, which can result from fibers of the sciatic nerve traveling through the piriformis muscle belly leading to impingement. We have determined that individuals with these structural variations in the thoracic outlet present with nTOS symptoms at a significantly higher rate than the general population, but that such anomalies are easily identified using ultrasonography.

Overall, nine of 44 of our student subject brachial plexuses were documented to have variant branching on US imaging, with the majority being the superior piercing variant. Four subjects presented with TOS secondary to a brachial plexus piercing variant. In each of these, the superior trunk or both the superior and middle trunks pierce the anterior scalene muscle (Figure 2 and Figure 4). Clinically, the superior piercing variant would cause neurologic symptoms in the C5 and C6 dermatomal distribution of the lateral arm, thumb, and second digit. Specifically, weakness and sensory deficits in the first two digits, and diminished reflexes of the biceps brachii and brachioradialis muscles [25,26,27,28]. The multiple piercing variant (Figure 2C) would result in more extensive neurogenic issues, corresponding with symptoms along the C5, C6, and C7 dermatomes, affecting the first through third digits of the hand. The clinical consequences could also include additional muscle weakness or decreased reflexes in the triceps brachii muscle [26,27,28].

One screening study participant and two cadavers were found to have *anterior variants*, with the superior trunk passing superficial to the anterior scalene muscle (Figure 6). This variation is less common than the piercing variants, and would not cause numbness or paresthesia in the hands or arms, but could render the nerve vulnerable to compression by forces such as those exerted by purses or backpacks. Impingement of the superior trunk, one of its proximal branches or the supraclavicular nerve is commonly known as pack palsy, which results from pressure on the shoulder girdle and is common in military personnel and hikers [29].

### 4.2. Diagnosis and Treatment of Thoracic Outlet Syndrome (TOS)

In the general population, primarily typical TOS has been clinically studied and is frequently diagnosed by vascular change with provocative testing. When a patient presents to a clinician with Atypical TOS, there is a potential for premature dismissal of TOS in the differential diagnosis. The diagnosis becomes increasingly elusive because the initial presentation and history of Atypical TOS correlate with other forms of neurologic impingement. Without proper identification of the etiology, the patient may not receive the most efficacious treatment. Based on the results of this study, with nearly half of reported TOS cases originating from variant anatomy which cannot be identified using provocative testing, it can be concluded that US may be a useful adjunct in clinical diagnosis. Ultrasonography is uniquely able to visualize variations and provide clinicians with an understanding of individual anatomy. By combining other diagnostic modalities, such as provocative tests, which can identify hypertonicity impingement, with US, clinicians would have the ability to visualize the structural composition of the neck and shoulder. The inclusion of such knowledge provides a higher level of diagnostic acuity when screening patients presenting with nonspecific TOS symptoms. Scalene blocks are another commonly performed diagnostic modality for nTOS, and can be less equivocal than provocative testing (e.g., [30]). However, these blocks may not be feasible in the primary care setting, and can leave patients with 2–36 h of residual discomfort or inconvenience following the procedure. US, on the other hand, is painless, rapid, and inexpensive, and can be easily implemented as part of a broader diagnostic approach. Due to the varied and complicated nature of nTOS presentation, it is often necessary for clinicians to apply multiple diagnostic modalities before ultimately arriving at a diagnosis. US can serve as an additional resource in the diagnostic toolkit of clinicians, especially in the primary care setting.

Utilizing this diagnostic approach it is also possible to tailor treatment to the individual’s unique anatomy. Treatment methods targeting either the first rib or scalene musculature would be complicated by the nerve branches entwined in the anterior scalene muscle belly. Surgical removal of rib 1 would likely not relieve the symptoms of compression around a more laterally placed trunk. A scalenectomy could place the piercing trunks in danger of damage unless appropriately identified [31]. Scalene botulinum toxin injection could be effective, so US could be applied to preselect patients with piercing variants for this treatment. For patients with one of the piercing variations, we propose a rational plan of osteopathic manipulative treatment (OMT) care and/or physical therapy consisting of indirect treatment modalities and the avoidance of direct techniques, based upon the potential for further impingement of the nerve within the muscle belly. This was evidenced in one subject suffering from atypical TOS resulting from a piercing variant [32]. In this case, the patient experienced optimal relief from her symptoms only when indirect treatment techniques were employed, reporting a significant improvement of her symptoms [32]. This patient also had improvement of her concurrent anxiety after gaining a more thorough understanding of her diagnosis with the US imaging.

## 5. Conclusions

Structural variations of the thoracic outlet, especially common brachial plexus branching variants, create a unique risk for neurogenic TOS that is difficult to diagnose clinically. Ultrasound is a reliable means of diagnosing this etiology when combined with provocative testing and patient history. Identification of these structural variants is crucial for developing an appropriate treatment plan, as certain types of current treatment modalities would be ineffective, or even exacerbate symptoms in patients with these variants.

## Figures and Tables

**Table 1:** Summary of standard provocative tests typically used to diagnose thoracic outlet syndrome and to rule out other upper extremity neurogenic conditions.

Provocative Test	Condition Tested	Description	Positive Test
TOS Tests			
Adson’s Test	Thoracic outlet syndrome (TOS)	Tests for compression of subclavian artery between anterior and middle scalene muscles. Monitor radial pulse with abduction, extension, and external rotation of upper extremity, and the head turned toward the affected side and then away.	Marked reduction of radial pulse or reproduction of symptoms
Costoclavicular Test	Thoracic outlet syndrome (TOS)	Tests for compression of subclavian artery between clavicle and first rib. Monitor radial pulse with patient forcefully hyper-retracting their scapulae.	Reduction of radial pulse
Hyperabduction/Wright Test	Thoracic outlet syndrome (TOS)	Tests for compression of subclavian artery by pectoralis minor muscle. Monitor radial pulse while holding the affected arm in a position of hyperabduction coupled with hyperextension.	Reproduction of symptoms or reduction of radial pulse
Rule-out Tests			
Carpal Compression Test	Carpal Tunnel Syndrome	Tests for impingement of the median nerve as it courses deep to the transverse carpal ligament. With wrist supinated, compress the carpal ligament.	Numbness and tingling within the median nerve distribution
Modified Spurling’s Test	Cervical root compression	Tests for cervical root compression at the cervical foramina. Patient’s head extended, ipsilaterally rotated, and ipsilaterally tilted with axial loading.	Reproduction of symptoms beyond shoulder blade

**Table 2:** Summary of cadaveric dissection results: quantification of anatomical variants in the relationship between the proximal brachial plexus and the scalene musculature.

Gender of Subjects	Classic Anatomy	C5 Anterior	Superior Piercing	Multiple Piercing	C5 Piercing
Male	13	2	29	4	3
Female	19	2	23	0	0
Total (%)	32 (33.7%)	4 (4.2%)	52 (52.7%)	4 (4.2%)	3 (3.2%)

**Table 3:** Brachial plexus variation in the screening sample, as identified by ultrasonographic evaluation.

Brachial Plexus Pattern	Frequency in Sample	% Symptomatic
Classic Anatomy	35; 79.5%	5; 13.9%
C5 Anterior Variant	1; 2.3%	0; 0%
Piercing Variants: Total	8; 18.2%	4; 50%
C5 Piercing Variant	1; 2.3%	1; 100%
Superior Piercing Variant	4; 9.1%	2; 50%
Multiple Piercing Variant	3; 6.8%	1; 33%

**Table 4:** Summary of findings of provocative testing and their association with self-reported neurogenic thoracic outlet syndrome (nTOS) symptoms across the entire screening sample.

Provacative Test Results and nTOS Symptoms	Adson’s Test	Costoclavicular Test	Hyperabduction/Wright Test
Positive test and reported nTOS symptomatic	3/16 (18.8%)	2/8 (25.0%)	3/13 (23.1%)
Negative test and reported nTOS asymptomatic	22/28 (78.6%)	29/36 (80.6%)	25/31 (80.6%)

## Supplementary Materials

Supplementary materials can be found at www.mdpi.com/2075-4418/07/3/40/s1.
